# Improving Asthma Guideline Implementation in Hospital Medicine (ImAGINE): A Single-site Improvement Initiative

**DOI:** 10.1097/pq9.0000000000000818

**Published:** 2025-06-12

**Authors:** Daniel Herchline, Michelle Mitchell, Shanon Brannen, Abbey Scott, Skyler Patterson, Sarah Khan, Katrina Sauerwein, Jennifer D. Treasure

**Affiliations:** From the * Department of Pediatrics, Cincinnati Children’s Hospital Medical Center, Cincinnati, Ohio; † Department of Pediatrics, University of Cincinnati College of Medicine, Cincinnati, Ohio; ‡ Department of Pediatrics, Children’s Health Plano, Plano, Texas.

## Abstract

**Introduction:**

The 2019 Global Initiative for Asthma (GINA) report called for changes in the approach to children with asthma. It focused on reducing morbidity and mortality and improving symptom control. This study aimed to increase the percentage of patients 6–18 years of age admitted for asthma exacerbations who received GINA guideline-concordant controller regimens from 39% to 50% within 6 months.

**Methods:**

Using the Model for Improvement framework, we garnered insight from pertinent stakeholders to identify key drivers. Key drivers identified included knowledge of guidelines, appropriate screening for symptom control, access to appropriate medications, and family and staff buy-in. Interventions initially focused on education around the GINA report. Subsequently, they implemented audit and feedback for prescribers, changes to standardized documentation (ie, asthma action plans and discharge instructions) in the electronic health record, and improved access to decision-support tools for prescribers through QR codes. An electronic health record query identified patients, and a control chart tracked study data weekly to determine the impact of interventions over time.

**Results:**

The average baseline concordance rate before interventions was 39%, with marked variability. A shift in the centerline occurred the month after the interventions began, with a final median centerline at 71%.

**Conclusions:**

This quality improvement study used a multifaceted approach to increase the number of patients discharged on a home maintenance regimen concordant with the most recent GINA report.

## INTRODUCTION

Asthma management for adult and pediatric patients has undergone a paradigm shift over the past decade, epitomized by the Global Initiative for Asthma (GINA) 2019 report.^1^ This report reviewed existing literature on the diagnosis and management of asthma and subsequently prioritized risk reduction and symptom control as 2 key drivers of the new recommendations. Among the major changes described in the report, the maintenance therapy approach for pediatric asthma patients represents one of the biggest evolutions for inpatient and outpatient pediatricians.^1^ These updates include 2 new evidence-based concepts: anti-inflammatory relievers and single maintenance and reliever therapy (SMART).^2,3^ These concepts are incorporated into new recommendations for initiating and adjusting maintenance therapy for patients with asthma. The recommendations are presented as stepwise tracks that aim to achieve optimal symptom control while minimizing the risk of exposure to oral corticosteroids and reducing short-acting beta-agonist use, both giving rise to adverse patient outcomes.^4,5^ The report places a strong emphasis on avoiding short-acting beta-agonist use as a monotherapy, given the associated risks of increased morbidity and mortality. To mitigate these risks, we report new strategies for treating pediatric patients with asthma, including needed low-dose inhaled corticosteroids during times of illness and the use of combined long-acting beta-agonist and inhaled corticosteroids (ICS-LABA) medications that are used as both daily controllers and relievers (SMART).^1^

### Rationale

At our institution, asthma management is variable. Although institutional clinical pathways exist, decision-making in lesser-defined practice areas varies from provider to provider, creating the potential for suboptimal patient care. Within our division of hospital medicine (HM), one facet of asthma practice that exhibited high variability involved the management of maintenance regimens for patients admitted with acute asthma exacerbations.

### Specific Aim

The project aimed to increase the percentage of patients 6–18 years of age admitted with asthma exacerbations and discharged from the HM service on GINA guideline-concordant controller regimens from 39% to 50% within 6 months. The team selected a goal of 50% given the vast number of stakeholders involved in enacting change, the anticipated challenges in promoting a large cultural shift, and the unknown impact of factors outside of institutional control, such as insurance coverage for controller medications. This intervention supports the overall goal of improving care for patients with asthma in our local community.

## METHODS

### Context

Our institution comprises a 684-bed pediatric quaternary-care center located in an urban setting, with a satellite campus 20 miles away. The satellite campus features 24 beds, with all subspecialties available in person or via telehealth, although it does not have a dedicated intensive care unit. Patient populations are like those at the base campus. The Division of HM has 77 physicians and 15 advanced practice providers. Most patients admitted to HM at both sites are cared for by teams of learners, including medical students and residents. All patients admitted with a diagnosis of asthma receive dedicated care from a respiratory therapist and a bedside nurse, both of whom contribute to providing asthma education. Our institution does not have asthma-specific clinical resources such as asthma navigators or specialized respiratory therapists (RTs). RTs are responsible for creating and reviewing asthma action plans for hospitalized patients based on the discharge prescriptions. Formal asthma education is provided with either an RT or bedside registered nurse at discharge.

### Improvement Team

A diverse, interprofessional team is involved in the care of asthma patients at our institution. Our team includes 2 respiratory therapists, a nurse, a clinical research coordinator, a nurse practitioner, and several physicians. We conceptualized and implemented a quality improvement initiative using the Model for Improvement to improve GINA-concordant care for patients admitted with asthma exacerbations. The team subsequently identified key stakeholders, which included nursing, respiratory therapy, social work, providers across multiple inpatient divisions involved in the care of patients with asthma (HM, pulmonology, allergy/immunology), residency program leadership, pharmacy, and outpatient primary care providers. Additionally, we met with family advocates to gain further insight into the experiences of patients and families and to identify potential opportunities and barriers. Our voice of the customer queries included these discussions with family advocates, which included family members of patients who had previously been admitted with asthma exacerbations, as well as formal surveys sent to nurses, respiratory therapists, and prescribers within the division of HM. (**See Supplemental file, Supplemental Digital Content 1**, which describes voice of the customer, https://links.lww.com/PQ9/A669.) These surveys included questions about perceived barriers to and opportunities for improving asthma care in the inpatient setting, particularly practices related to adhering to the GINA recommendations.

**Fig. 1.**
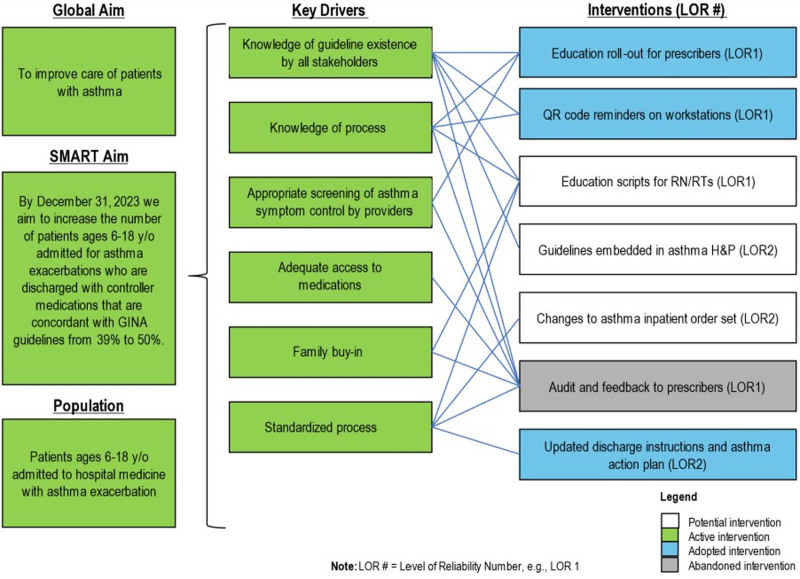
Key driver diagram. LOR #, level of reliability number, for example, LOR 1.

To better conceptualize the problem of interest, we analyzed the root cause using improvement tools, including a key driver diagram, process map, and customer voice. We crafted the key driver diagram with guidance from improvement experts as part of a formal improvement science course, drawing on the perspectives of all team members following the development of a process map and simplified failure mode effects analysis (**See Supplemental file, Supplemental Digital Content 2**, which describes process map, https://links.lww.com/PQ9/A670.) (**See Supplemental file, Supplemental Digital Content 3**, which describes simplified failure mode effects analysis, https://links.lww.com/PQ9/A671.) We identified 6 primary drivers: (1) knowledge of GINA guidelines, (2) knowledge of existing institutional processes, 3) appropriate screening of symptom control by providers, (4) adequate access to medications, (5) family buy-in, and (6) standardizing clinical care processes (Fig. 1).

### Interventions

Interventions primarily focused on increasing knowledge of GINA guidelines and standardizing clinical care processes. Interventions included project announcements with rationale, staff education, provider audit and feedback, and electronic health record modifications.

#### Project Announcement

The first intervention consisted of a straightforward divisional announcement with a summary of the project’s goals and logistics. In this announcement, we included educational information about the GINA recommendations and reviewed data on key metrics as part of the primer.

#### Staff Education

The second intervention involved the rollout of educational materials to all team members involved in the care of patients admitted with asthma, including nursing, respiratory therapy, resident physicians, and members of the Division of HM. The research team created and distributed role-specific educational materials to the appropriate providers and conducted formal didactics with the residents and HM providers. Education focused on highlighting new information from the GINA report, the rationale for changing the approach to asthma care, and specific logistics for prescribing medications per the GINA recommendations. Educational materials included just-in-time training documents with instructions on how to step up controller regimens, appropriate dosing, and Medicaid coverage options within our geographical region. More expansive tools covered evidence and rationale from the GINA report and guidance around providing education to families. The team housed educational toolkits online in multiple locations and physically placed toolkits in all resident and HM provider workrooms for just-in-time use. The team placed QR codes linked to the educational materials on all resident mobile workstations to increase access during family-centered rounds. (**See Supplemental File, Supplemental Digital Content 4**, which describes just-in-time education materials, https://links.lww.com/PQ9/A672.)

#### Provider Audit and Feedback

The third intervention utilized audit and feedback for HM providers. At the beginning of each week, all providers on service for the week received an email reminder that included the educational toolkit and the project’s goals. At the end of the week, a subsequent email provided feedback on clinical decision-making regarding GINA recommendations and solicited feedback on improving implementation. Specific instances where providers did not adhere to recommendations represented primary points of emphasis for feedback emails. These emails included a request to provide additional insight into clinical decision-making, particularly if the electronic health record documentation was unclear.

#### Electronic Health Record Modifications

Finally, the team adjusted discharge instructions within the electronic health record to reflect updated GINA recommendations. These modifications to discharge instructions included options for GINA-concordant care but did not represent true clinical decision-support interventions. Rather, these changes in language within the templates served as a “nudge” to providers, making it more difficult for them to stray from guideline recommendations. For example, the language surrounding rescue medications was changed from “albuterol” as the default to require providers to list the specific medication for rescue therapy if they chose SMART for a patient’s home regimen.

### Study of the Interventions

We collected data on all patients admitted to the HM service diagnosed with an acute asthma exacerbation. The team collectively developed the process for identifying eligible patients, agreed on the important patient data to collect, and discussed decision-making regarding determining guideline concordance. We reviewed a subset of eligible patients as a team during baseline data collection, and Daniel Herchline conducted subsequent chart reviews during the preintervention, intervention, and postintervention periods. The team extracted eligible patients using billing codes and confirmed the diagnosis by verifying the discharge summary. Data analysis included patients with multiple diagnoses listed in the discharge summary, provided that asthma exacerbation was one of the diagnoses. The analysis excluded patients, however, if they had a singular nonasthma diagnosis (eg, viral-induced wheeze, community-acquired pneumonia) listed in the discharge summary.

We collected weekly data for 4 months before the first study intervention (“preintervention” period: June 2023–September 2023), for 3 months during study interventions (October 2023–December 2023), and for 8 months following the interventions (“postintervention” period: January 2024–August 2024). We decided on a longer postintervention period to ensure sustained progress through the beginning of a new academic year, including learner turnover and new faculty hiring. We performed individual chart reviews for all patients deemed eligible for inclusion. Collected data included the patient’s maintenance regimen at admission, documentation of asthma severity and degree of control (if available), and notes on factors that impacted medical decision-making, such as access to care and medication adherence. Additionally, data were collected on subspecialty consultants involved in asthma management during admission and the maintenance regimen at discharge. The team then designated each patient’s discharge maintenance regimen as either concordant with the GINA recommendations or not concordant. A small subset of patient families deferred any change in therapy to their primary outpatient provider, who was in charge of asthma management. These patients were still classified according to concordance with GINA recommendations based on asthma control and severity. However, we acknowledge that these cases often represent a commitment to family-centered care rather than a true failure to adhere to the recommendations. In addition to designating GINA concordance, we also noted whether maintenance therapy was “stepped up” at discharge.

### Measures

The primary outcome measure was the percentage of patients discharged from the hospital on GINA-concordant maintenance regimens. A secondary outcome of interest was the percentage of patients whose controller regimens were stepped up at the time of discharge, a measure chosen to help contextualize the data and differentiate errors made in discharge prescribing. Balancing measures included the median length of stay (LOS) for patients admitted with asthma exacerbations to the HM service and the 30-day readmission rate for patients admitted with asthma exacerbations.

### Data Analysis

An statistical process control chart analyzed the primary outcome measure using standard rules to determine special cause variation.^6–10^ A Wilcoxon rank-sum test was used to compare the median LOS before and after intervention. The chi-square test compared pre- and postintervention 30-day readmission rates. Additionally, we plotted both balancing measures on run charts.

### Ethical Considerations

This project was undertaken as a quality improvement initiative and does not constitute human subjects research.

## RESULTS

**Fig. 2.**
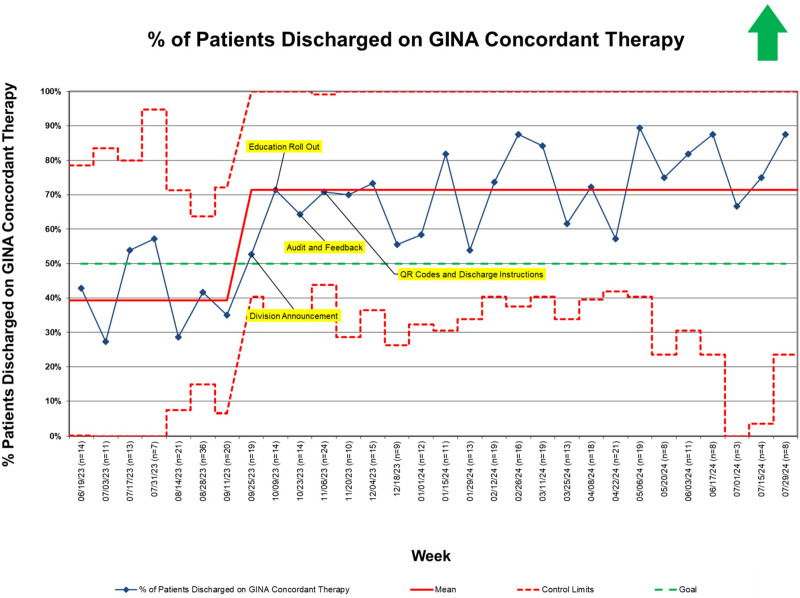
SPC chart showing percentage of patients discharged on GINA-concordant therapy. SPC, statistical process control.

**Fig. 3.**
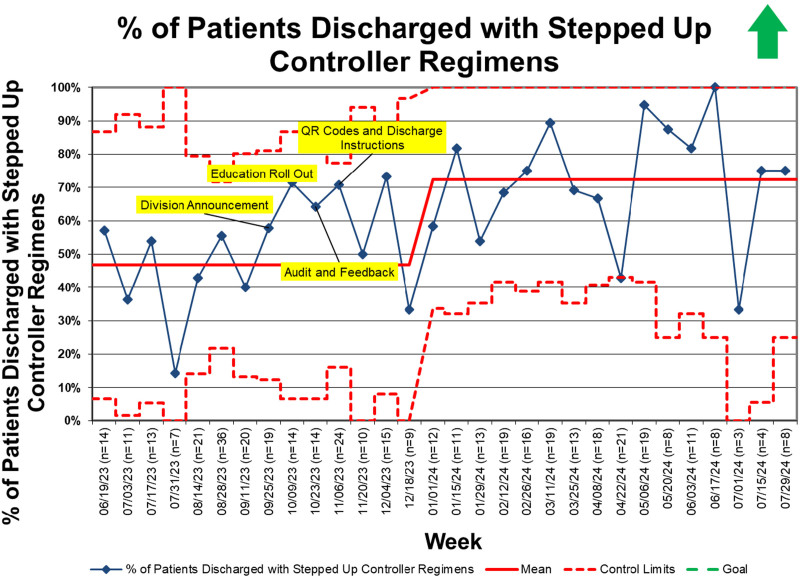
SPC chart showing percentage of patients discharged with stepped-up controller regimens. SPC, statistical process control.

A total of 436 patients (122 preintervention, 105 during the intervention period, and 209 postintervention) met the inclusion criteria during the study period. During the preintervention period, clinicians prescribed controller medications that adhered to GINA recommendations in 39% of patients. The percentage of patients discharged with guideline-adherent regimens increased following the first improvement cycle. It reached special cause variation (8 or more points above the centerline) during the intervention period (Fig. 2). We established a new centerline of 71% and sustained this change until project completion. Decisions to step up the controller regimen during admission followed similar trends (Fig. 3).

**Fig. 4.**
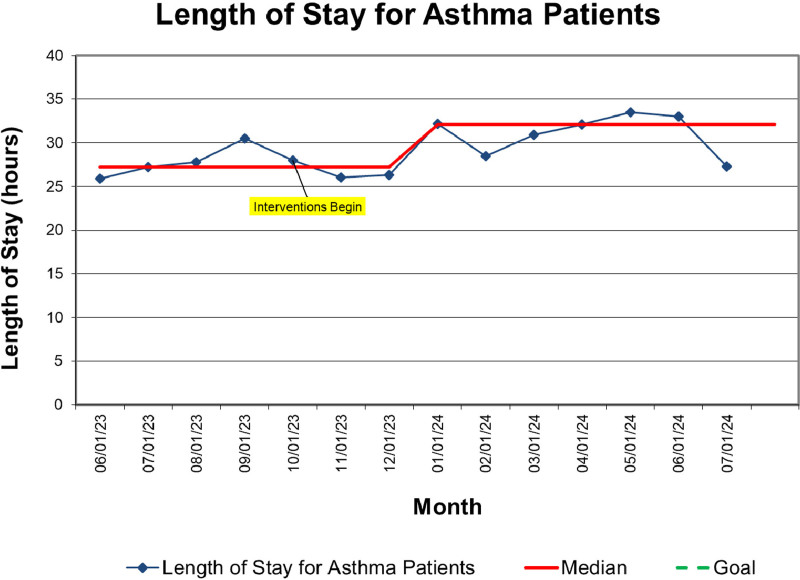
Run chart showing LOS.

**Fig. 5.**
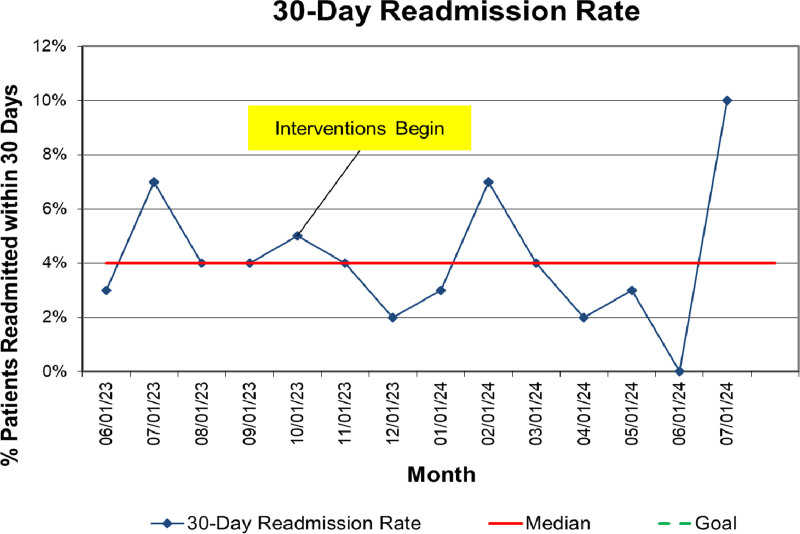
Run chart showing 30-day readmission rates.

Balancing metrics revealed a slightly lengthened median LOS for patients (27.2 h preintervention, 30.1 h at the end of the postintervention period), with no increase in 30-day readmissions, which remained stable at a median of 4% (Figs. 4, 5).

Our division adopted each study intervention except for provider audit and feedback, which ceased at the end of the intervention period. All educational materials remain available for provider reference.

## DISCUSSION

Using quality improvement methodology, we increased the number of patients with asthma discharged on controller regimens concordant with GINA recommendations without significantly increasing the LOS or readmission rates. Our approach leveraged several techniques that have proven successful in guideline implementation, including broad stakeholder engagement, preidentification of barriers, leveraging standardized pathways, and use of a conceptual framework in the Model for Improvement.^11–14^ Although it is difficult to determine which interventions had the greatest impact, given the timing of implementation, we suspect that, in alignment with prior research, our educational outreach and audit/feedback interventions likely had a marked effect.^15^ Interestingly, we observed an immediate increase in the number of patients discharged on guideline-concordant regimens following the announcement of the study to HM faculty. Although we attempted to craft interventions that would impact all providers, we noted persistent variability in adherence to the guidelines and the decision to step up therapy during admission.

Despite abundant evidence to support practice change, producing a cultural shift among all providers proved challenging. Within our institution, multiple specialties are responsible for caring for patients with asthma. Although multiple collaborative groups work to align our overall institutional approach to caring for patients with asthma, occasional differences in management styles across subspecialties may have contributed to varied practice among trainees and other interprofessional care team members. The concepts of using a combined ICS-LABA as both maintenance and reliever therapy, as well as using as-needed ICS for certain patients, represented the biggest deviations from institutional practice, as reported by stakeholders during the study period. This challenge underscored the importance of considering organizational culture when developing targeted implementation strategies.^16,17^ In alignment with organizational values, our efforts aimed to balance patient safety, value, and the patient/family experience. Across all our interventions, we focused on providing family-centered care that emphasizes shared decision-making and effective communication with families regarding asthma management decisions to optimize health literacy, which is known to impact asthma outcomes.^18^ Our multidisciplinary educational approach sought to provide all members of the interprofessional care team with the necessary tools to discuss these new concepts with patients.^19^ This included adjustments to the asthma action plans provided to patients at discharge, which our institution’s respiratory therapists extensively use for patient education.

Another predicted barrier to implementation revolved around the complexities of formulary coverage for private insurers and Medicaid across multiple states.^20^ To assist prescribers, we provided a list of approved ICS-LABA for each state’s respective Medicaid within our geographical region. To align with our institution’s prior practice, we sent all patients home with at least two controller inhalers: one for home use and one for school use. We acknowledge that this may still be suboptimal for patients who spend time in multiple households but were largely unsuccessful in providing more than 2. Embedded in the education and audit/feedback interventions, we asked providers to give feedback on any challenges they experienced in prescribing controller medications. We also engaged in outreach to community pediatricians to solicit feedback in this realm, but we recognized that our inability to accurately capture failures after discharge was a notable limitation.

Although they show similar trends, it is worth noting the differences between the percentage of patients discharged home on GINA-concordant regimens compared with the percentage that had their home regimen stepped up during hospitalization. A few drivers explain this phenomenon. Firstly, providers frequently resumed patients’ prior home regimens without a step up in instances of suboptimal medication adherence unless the family noted uncontrolled symptoms despite appropriate use of prescribed medications. Despite the regimen remaining unchanged, we considered these guideline-concordant regimens if their prior regimen was deemed acceptable according to the guidelines. We made this decision to both achieve symptom control and minimize side effects. Additionally, several families chose not to change home regimens despite provider recommendations. In striving to balance optimal symptom control and patient-centered care, providers occasionally opted not to make changes if the family preferred their primary outpatient asthma provider to direct care or if the family reported significant hesitation about changes to the patients’ home regimens. Finally, there were instances in which providers did step up the regimen during admission but did not select an appropriate step based on the documented asthma severity and symptom control. Most frequently, providers bypassed as-needed ICS-LABA for older patients despite reported mild symptoms.

The study had several important limitations. As previously mentioned, the clustering of interventions makes it difficult to assess the impact of each intervention. Second, during chart review, it was typically not difficult to determine the appropriateness of the discharge controller regimen based on documented asthma severity, symptom control, and other contributing factors. However, in a limited number of cases, it was not easy to adjudicate the appropriateness of the discharge regimen due to inadequate documentation, confusion regarding prior home regimens, and an inability to characterize contributing factors such as family-centered care and medication coverage limitations. A prior study similarly described these difficulties.^21^ Third, we did not create an extensive sustainability plan because a separate group within our institution initiated a new improvement project with significant overlap shortly following the completion of our study. The new study aimed to improve several metrics for patients with asthma, one of which was discharge on an appropriate regimen for all patients admitted with asthma exacerbations. Given the aims of the new study, we did not feel that we could separate the impact of our interventions from those of the new study, which includes feedback to providers. However, we believe that the new study will continue to improve our work and is part of a larger sustainability plan. Finally, our institution has an expansive infrastructure for supporting ongoing patient safety and improvement work, which may limit generalizability.

## CONCLUSIONS

This multifaceted initiative combined quality improvement and interprofessional education to increase the number of patients admitted with asthma exacerbations who were discharged home on guideline-concordant controller regimens. Despite the encouraging results of this study, further work is needed to ensure that patients with asthma can achieve optimal symptom control.

## Supplementary Material


