# Supplementary material for: Improving Asthma Guideline Implementation in Hospital Medicine (ImAGINE): A Single-site Improvement Initiative
Source: Pediatr Qual Saf. 2025 Jun 12;10(4):e818. doi: 10.1097/pq9.0000000000000818 (PMC12160742; doi:10.1097/pq9.0000000000000818)
Supplement: Supplementary file 1 [file pqs-10-e818-s001.pdf]

Voices of the Customer

Revision Date: *9/6/2023 (v1)*

Open-Ended Question:

	Customer 1: Patient/Family	Customer 2: RN/RT	Customer 3: MD/APP
**Voice of the Customer (VOC)**<br>Customer Feedback Section – Verbatim<br>What does the customer say?	"Can there be instructional Youtube video for discharge?"<br>"I'm definitely in favor of changes if it is best for my child."	"Looking forward to continuing evidence-based care, especially if it helps ease our staffing."<br>"I worry about families being confused and about insurance issues."<br>"I think this could help limit asthma admissions."	"I'll do whatever evidence says is best for my patient."<br>"All changes should definitely be communicated to PCPs."<br>"I'm a little skeptical about changes being an asthmatic myself."
**Critical To –**	[x] Outcomes [x] Safety<br>[x] Efficiency [x] Equity<br>[x] Experience [x] Value	[x] Outcomes [x] Safety<br>[x] Efficiency [ ] Equity<br>[ ] Experience [ ] Value	[x] Outcomes [x] Safety<br>[x] Efficiency [ ] Equity<br>[ ] Experience [ ] Value
**Customer Requirements**<br>•What's important to the customer?<br>•What's the customer's experience and expectation ?<br>•What are the key requirements to addressing the problem statement / global AIM?	Patient-centeredness is of the utmost importance and families were supportive of changes if they lead to improved outcomes for their children.<br>Thinking about ways to make care easier for families and more clear communication were critical.	RNs/RTs were excited about the changes as they will hopefully decrease the number of admissions and would improve symptom control for patients which could ease burdens on staffing. There were some concerns about educating parents on the new changes and how they would be accepted.	MD/APPs overall in favor of the changes as they are evidence-based but had a number of concerns about adherence, logistics, and cultural shift.

Supplemental File. Voice of the Customers.
