# Supplementary figures and images for: Improving Asthma Guideline Implementation in Hospital Medicine (ImAGINE): A Single-site Improvement Initiative
Source: Pediatr Qual Saf. 2025 Jun 12;10(4):e818. doi: 10.1097/pq9.0000000000000818 (PMC12160742; doi:10.1097/pq9.0000000000000818)

# Asthma Admission Process Map

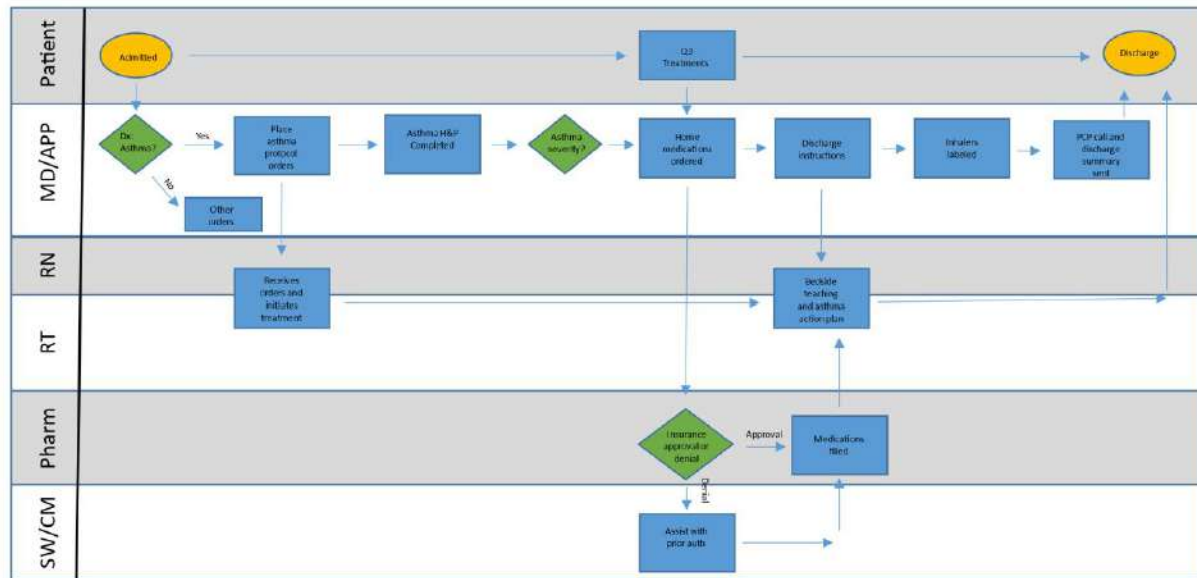

Supplemental File: Asthma Admission Process Map

Supplement: Supplementary file 2 [file pqs-10-e818-s002.pdf]

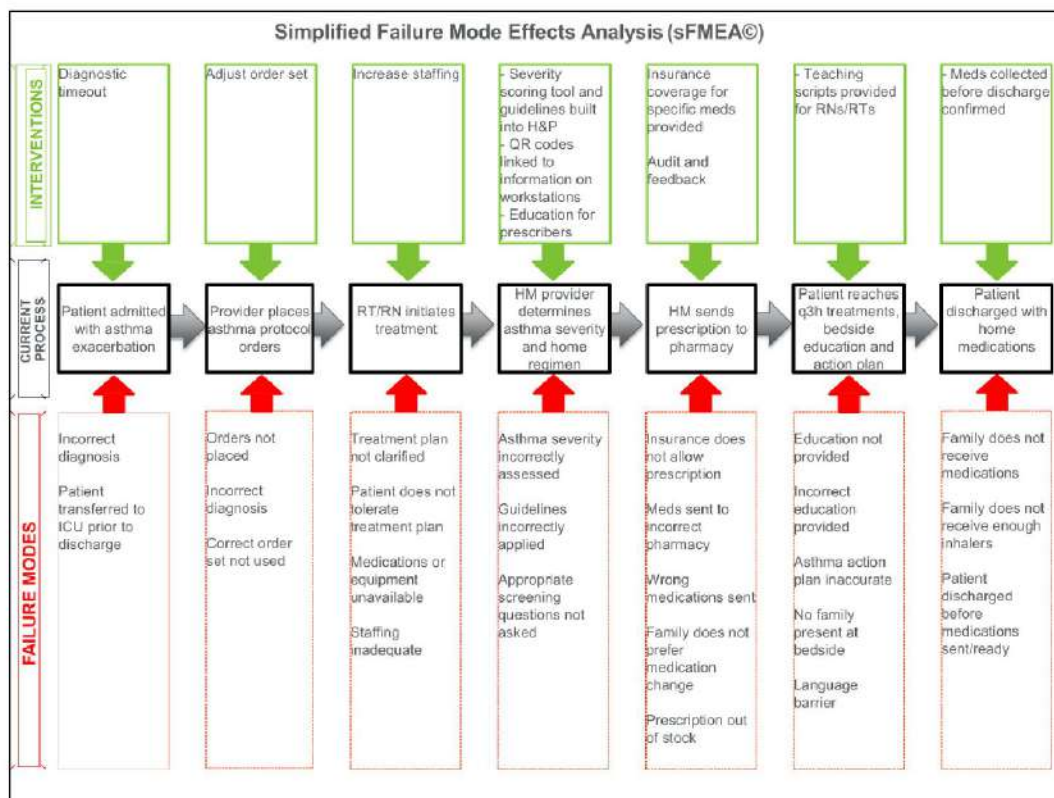

**Supplemental Figure. Simplified Failure Mode Effects Analysis (sFMEA).**

Supplement: Supplementary file 3 [file pqs-10-e818-s003.pdf]
