# Supplementary material for: Improving Asthma Guideline Implementation in Hospital Medicine (ImAGINE): A Single-site Improvement Initiative
Source: Pediatr Qual Saf. 2025 Jun 12;10(4):e818. doi: 10.1097/pq9.0000000000000818 (PMC12160742; doi:10.1097/pq9.0000000000000818)
Supplement: Supplementary file 4 [file pqs-10-e818-s004.pdf]

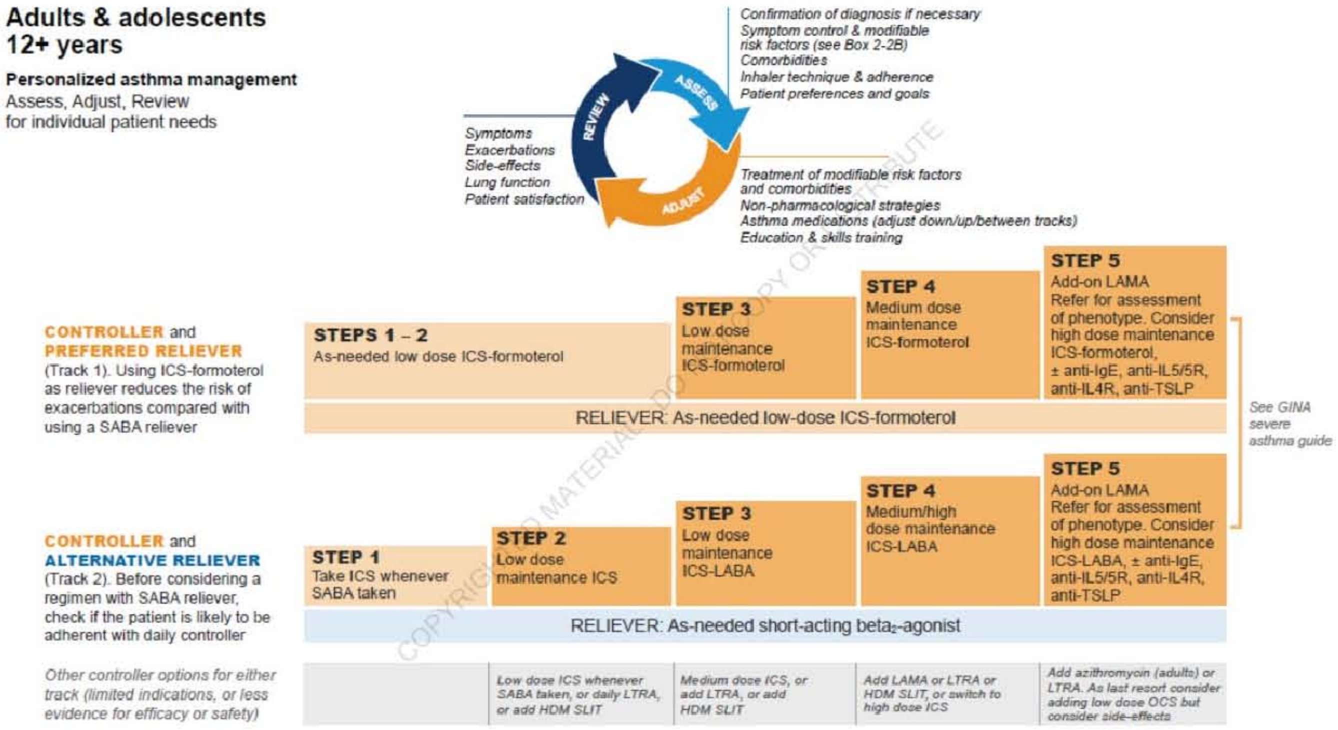

HDM: house dust mite; ICS: inhaled corticosteroid; LABA: long-acting beta$_2$-agonist; LAMA: long-acting muscarinic antagonist; LTRA: leukotriene receptor antagonist; OCS: oral corticosteroids; SABA: short-acting beta$_2$-agonist; SLIT: sublingual immunotherapy. For recommendations about *initial* asthma treatment in adults and adolescents, see Box 3-4A (p.55) and 3-4B (p.56). See Box 3-6, p.63 for low, medium and high ICS doses for adults and adolescents.

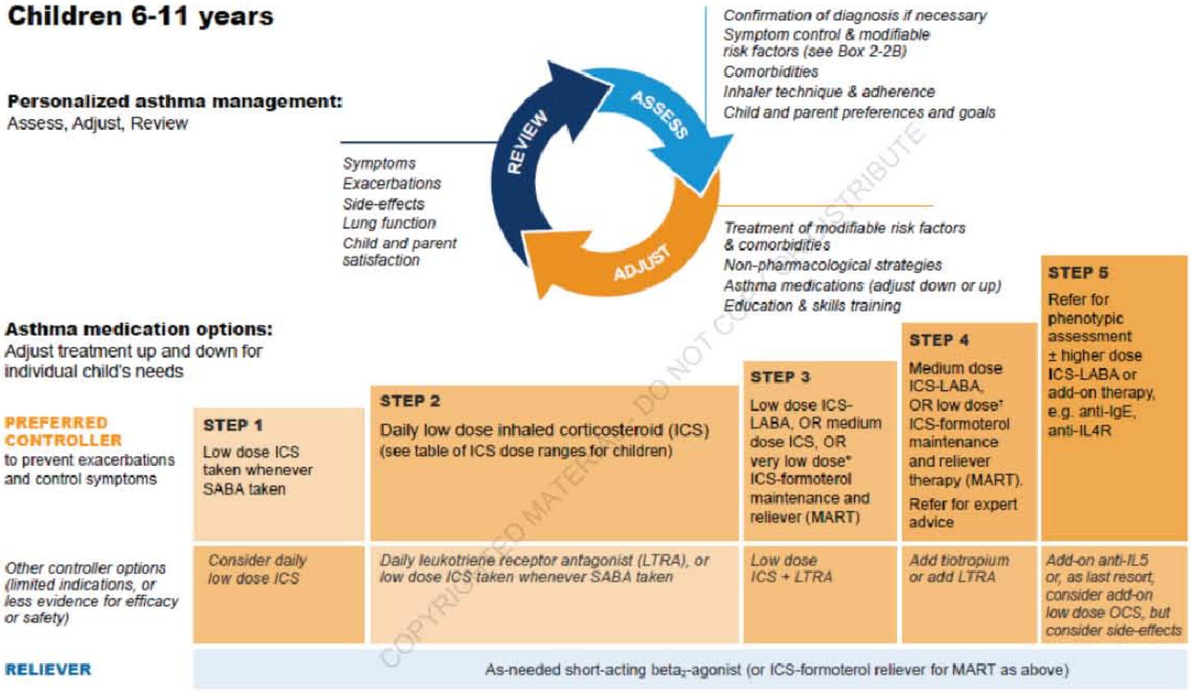

*Very low dose: BUD-FORM 100/6 mcg
†Low dose: BUD-FORM 200/6 mcg (metered doses).

BUD-FORM: budesonide-formoterol; ICS: inhaled corticosteroid; LABA: long-acting beta$_2$-agonist; LTRA: leukotriene receptor antagonist; MART: maintenance and reliever therapy with ICS-formoterol; OCS: oral corticosteroids; SABA: short-acting beta$_2$-agonist. For *initial* asthma treatment in children aged 6–11 years, see Box 3-4C (p.58) and Box 3-4D (p.59)
See Box 3-6, p.63 for low, medium and high ICS doses in children.

[redacted]
